# Use of Transglutaminase 2 mRNA expression in peripheral blood mononuclear cells in patients with Radiologically Isolated Syndrome as a neuroinflammation biomarker: A preliminary study

**DOI:** 10.3934/Neuroscience.2025015

**Published:** 2025-06-24

**Authors:** Rosa Giacca, Miriana Conte, Alessandro d'Ambrosio, Alvino Bisecco, Renato Docimo, Mario Risi, Manuela Altieri, Riccardo Borgo, Rosario Domenico Melisi, Vittorio Gentile, Antonio Gallo

**Affiliations:** 1 Department of Precision Medicine, University of Campania “Luigi Vanvitelli”, Naples, Italy; 2 First Division of Neurology and Neurophysiopathology, AOU “Luigi Vanvitelli”, Naples, Italy; 3 Department of Advanced Medical and Surgical Sciences, University of Campania “Luigi Vanvitelli”, Naples, Italy; 4 Interuniversity Center of Neuroscience Researches (CIRN), University of Campania “Luigi Vanvitelli”, Naples, Italy

**Keywords:** transglutaminase 2, multiple sclerosis, radiologically isolated syndrome, neuroinflammation, biomarker

## Abstract

The calcium-dependent enzyme Transglutaminase 2 (TG2) (E.C. 2.3.2.13), which can promote post-translational modifications of proteins, is involved in several physiological processes, including development, neuronal cell death, and differentiation, as well as synaptic plasticity and transmission in the central nervous system (CNS). Several studies highlight the potential role of the TG2/NF-κB activation pathway in neurodegenerative diseases, including Multiple Sclerosis (MS), and the neuroinflammation that is associated with these conditions. The cross-linking activity of TG2, facilitating the formation of isopeptide bonds between glutamine and lysine residues, appears to be involved in forming protein aggregate deposits in these pathological conditions. Specifically, in the chronic neuroinflammation of MS, TG2 seems to play a central role in the fibrotic process of the lesion. Several potential biomarkers have been investigated for the prognosis and monitoring of MS, but no researchers have explored the presence of potential inflammatory signals in peripheral blood mononuclear cells (PBMCs) during the presymptomatic stage of MS, known as Radiologically Isolated Syndrome (RIS), on account of the lack of information regarding its pathological aspects. Since researchers have demonstrated a correlation between TG2 mRNA levels in PBMCs and the clinical and radiological progression of MS, we aimed to evaluate the expression levels of TG2 in RIS patients, comparing them with those in relapsing-remitting MS (RRMS) patients and healthy controls (HCs) using real-time PCR analysis. Preliminary data showed that RIS patients exhibit lower TG2 mRNA expression levels compared to RRMS patients, while no difference in TG2 mRNA expression being observed between RIS patients and HCs. This suggests that RIS patients exhibit a lower neuroinflammation grade than RRMS patients and that TG2 may represent a potential biochemical marker for assessing neuroinflammation associated with this disease. Future investigations may include longitudinal assessments of the potential role of TG2 mRNA blood levels in predicting or monitoring the progression from RIS to MS.

## 1. Introduction

In multiple sclerosis (MS), there is a strong need for an accessible and reliable serum biomarker that supports the diagnosis, prognosis, and monitoring of the disease. Among other potential MS serum biomarkers, TG2 (E.C. 2.3.2.13), a calcium-dependent enzyme involved in several physiological processes in the nervous system, functions in neurodegenerative (e.g., Alzheimer's disease) and neuroinflammatory diseases [1]. In animal models of MS, TG2 expression in blood-derived leukocytes has been linked to monocyte infiltration into the CNS [2]. More recently, PBMC-derived TG2 mRNA levels were significantly associated with MS clinical and radiological progression [3], thus supporting further investigations regarding the role of TG2 expression as a potential biomarker for disease progression in patients with MS. Radiologically Isolated Syndrome (RIS) is defined by MRI findings meeting the McDonald criteria [4] for MS in the brain and/or spinal cord in asymptomatic patients. In this group of patients, a MRI scan typically demonstrates incidental white matter lesions characteristic of MS in location and morphology, but without a clinical history of demyelinating attack or ongoing neurologic deterioration, as well as other alternative cause of white matter damage such as vascular, infectious, or drug-induced (toxic) etiologies [5]. Radiological progression usually occurs in approximately 2/3 of patients with RIS, while 1/3 of patients over a 5-year period may subsequently develop neurological symptoms that enable a definite diagnosis of MS [6]. RIS shares the same epidemiology and risk factors of MS and represents an emerging field of investigation of presymptomatic MS. In 2025, RIS criteria were updated by Lebrun-Frenay et al. [6] with the presence of cerebrospinal fluid oligoclonal bands (CSF OCBs) remaining the most reliable biological marker of chronic (i.e., dissemination in time) inflammatory and autoimmune demyelination of the CNS. Accordingly, the presence/number of CSF OCBs is a strong predictor of conversion to MS [7]. Nevertheless, even if CSF OCBs have a very high sensitivity and specificity for MS diagnosis, sensitivity does not reach 100%, and collection of CSF via lumbar puncture remains an invasive procedure. Thus, our goal was to confirm the overexpression of TG2 in RRMS by exploring TG2 expression in RIS compared to RRMS and healthy controls (HCs).

## 2. Materials and methods

### 2.1. Recruitment of the study cohort

In this study, a total of 10 RIS and 10 RRMS patients were consecutively recruited from the I Neurology Clinic of the Department of Advanced Medical and Surgical Sciences of the University of Campania Luigi Vanvitelli. Inclusion criteria for patients for RIS and RRMS patients were: a) confirmed diagnosis of MS according to McDonald 2024 criteria for RRMS patients [4] and Lebrun-Frenay 2025 [6] revised diagnostic criteria for RIS patients; b) no disease-modifying treatment at the time of recruitment; c) age ≥ 18 years; d) no history of other neurodegenerative or inflammatory conditions; and e) ability and willingness to sign an informed consent form. Disability was scored at the baseline visit using the Expanded Disability Status Scale (EDSS). Inclusion criteria for HCs were: a) age ≥ 18 years; b) no history of neurodegenerative or inflammatory conditions; c) absence of ongoing therapeutic treatment; and d) ability and willingness to sign an informed consent form. Additional data of biological parameters such as quantification of oligoclonal bands (BOC), Kappa-index and CSF IgG- index and radiological parameters (i.e., the white matter lesions volume) from MRI scans were collected for all patients.

### 2.2. Isolation of PBMCs from human peripheral blood

Isolation of PBMCs from human peripheral blood was carried out using a protocol described by Sestito et al. [3], as follows. Blood was collected in a tube containing the anticoagulant Ethylenediaminetetraacetic acid (EDTA) and diluted with an equal volume of culture medium RPMI-1640 w/o FBS. Ficoll was added to the centrifuge tube. Using a pipette, diluted blood was carefully layered over the Ficoll. Since Ficoll has a higher density than the cell suspension, a distinct interface was formed. The samples were centrifuged at room temperature for 20 minutes at 2000 rpm. After centrifugation, a buffy coat containing PBMCs was formed at the interface. Using a Pasteur pipette, the cells at the interface were carefully removed and transferred to a new centrifuge tube. The transferred cells were diluted with a medium to reduce the density of the solution. After another centrifugation at 1200 rpm for 5 minutes, the supernatant was discarded, and the pellet, consisting of PBMCs, was retained. The PBMCs were washed 2–3 times in medium (a serum-containing medium can be used at this stage) before proceeding to further treatment.

### 2.3. RNA extraction and quantitative Real Time -PCR analysis

RNA extraction from cell cultures was performed using the Chomczynski and Sacchi protocol [8] utilizing TRI®Reagent (Sigma). The quality and concentration of RNA were assessed by spectrophotometry by the NanodropTM instrument and RNA electrophoresis in denaturing conditions. The reverse transcription was performed using the 5X All-In-One RT Master Mix (abm) according to the manufacturer's instructions. Semi-quantitative Real Time-PCR analyses were carried out on 50–100 ng DNA-free RNA. The reaction volume mixture was 10 µL and each primer (Table 1) was used at a final concentration of 300 nM. Plates were run using the BioRad CFX96 Real-Time PCR detection system (Bio-Rad Laboratories Inc.) and each set of both a non-reverse transcription control and a no template sample negative control were included. The reaction kit contained SYBR green as detection system. The thermocycling protocol consisted of an activation cycle of 2 min at 95°C, then 40 cycles of a denaturation step for 5 s at 95°C, an annealing/extension step for 30 s at 60°C, and an extension step at 72°C. Relative changes in gene expression were quantified using the comparative Ct (ΔCt, ΔΔCt) method as described by Livak and Schmittgen [9]. The Ct values of the genes of interest were normalized to an average of the endogenous housekeeping genes GAPDH and B-Actin.

**Table 1.** Primers used.

**Human Gene**	**Forward**	**Reverse**
GAPDH	5′-GGGCATCCTGGGCTACACTGAGCACC-3′	5′-GGGGGACTGAGTGTGGCAGG-3′
Β-Actin	5′-TTGTTACAGGAAGTCCCTTGCC-3′	5′-ATGCTATCACCTCCCCTGTGT-3′
TG2	5′-CCTTACGGAGTCCAACCTCA-3′	5′-CCGTCTTCTGCTCCTCAGTC-3′

### 2.4. Statistical analysis

Experiments were performed at least three times with replicate samples. Data are expressed as mean ± standard deviation (SD). The means were compared using analysis of variance (ANOVA) plus post hoc Tukey's Test. A p-value of <0.05 was considered to indicate a statistically significant result.

## 3. Results

**Figure 1.**
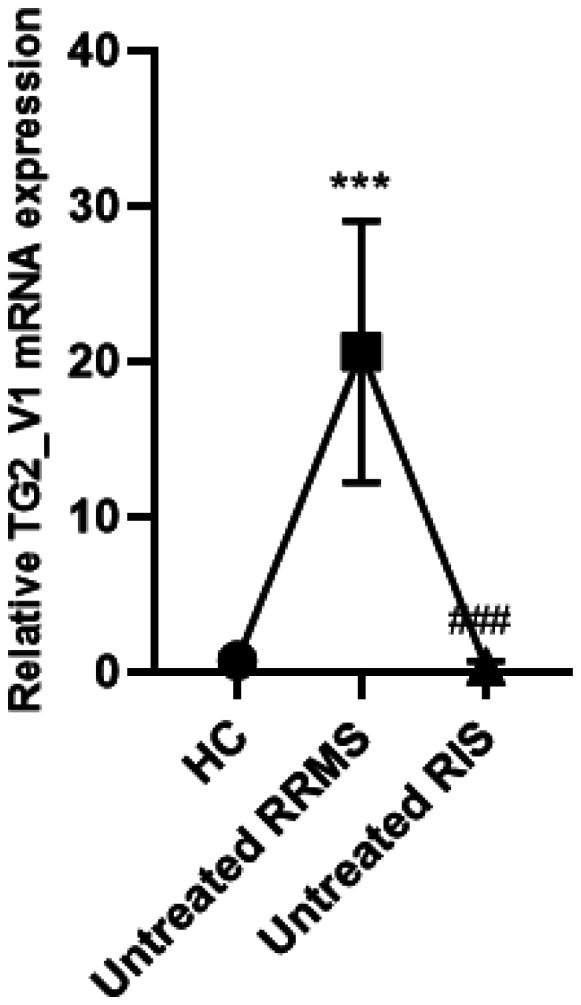
Real Time-PCR analyses were performed to measure the mRNA expression of TG2 in human monocytes from RRMS (n = 10) and RIS patients (n = 10), and HCs (n = 10). Data are shown as the mean ± SD of three independent experiments. RRMS > HC, ***p < 0.001; RRMS > RIS, ^###^p < 0.001; RIS vs. HCs (no significant). One-way ANOVA followed by Tukey's post hoc test.

Preliminary data showed that RIS patients exhibited TG2 mRNA expression levels at baseline comparable to healthy controls, suggesting basal levels of neuroinflammation. On the contrary, high levels of TG2 mRNA expression were observed in RRMS patients. Thus, these data suggest that RIS patients exhibit a basal neuroinflammation grade in comparison with RRMS patients (Figure 1).

## 4. Discussion and conclusions

In this study, we aimed to further explore the role of TG2 as a possible biomarker of MS pathophysiology in RRMS and, for the first time, in presymptomatic MS (i.e., RIS). We confirmed that TG2 expression is higher in RRMS as compared to HCs, but, most interestingly, we observed that TG2 expression in RIS is comparable to HCs and significantly lower than RRMS. These results suggest that TG2 expression levels might reflect the clinical stage of the disease, thus reinforcing the need to further investigate TG2 as a potential biomarker for disease staging, monitoring, and prognosis. Another promising biomarker emerging in MS is represented by the serum levels of Neurofilament light chains (NfLs). However, serum NfLs testing is expensive and suffers from several confounding variables such as age, body mass index (BMI), and renal functions [10]. Most importantly, serum NfL levels are an indicator of neuronal damage and, therefore, are not specific to MS. Indeed, elevated serum NfL levels do not differentiate MS patients from those with infections or other neurological diseases. This highlights the need to search for an MS-specific marker. It has been shown that TG2 is highly sensitive to various activators, including NF-κB. Given its role in inflammatory processes and neurodegenerative diseases, TG2's involvement in neuroinflammation has been studied, particularly in the context of microglial cells and astrocytes changes related to CNS damage. Altered levels of TG2 mRNA have been described in PBMCs derived from MS patients [3] and have been associated with disease evolution. Our study, therefore, confirms this previous study, which correlated TG2 mRNA expression levels in PBMCs with clinical and radiological progression of MS. Moreover, the analysis of TG2 mRNA expression levels in the preclinical phase, i.e., RIS, goes further than the exploration of this biomarker in the context of MS disease spectrum and might help in defining the neuroinflammatory status of these patients and predicting their proximity to conversion to MS. Future longitudinal studies need to explore the timing of TG2 expression rise before conversion from RIS to MS and the correlations between TG2 expression levels and radiological activity (i.e., appearance of new MRI lesions), alongside lesion load and other soluble biomarkers such as serum NfLs. It would also be of interest to evaluate the correlation in RIS and RRMS, between TG2 levels, lesion load, and subsequent clinical and radiological progression.

## Use of AI tools declaration

The authors declare they have not used Artificial Intelligence (AI) tools in the creation of this article.
